# Fixed-Effects Analyses of Time-Varying Associations between Hobbies and Depression in a Longitudinal Cohort Study: Support for Social Prescribing?

**DOI:** 10.1159/000503571

**Published:** 2019-10-28

**Authors:** Daisy Fancourt, Simon Opher, Cesar de Oliveira

**Affiliations:** ^a^Department of Behavioural Science and Health, University College London, London, United Kingdom; ^b^May Lane Surgery, Gloucestershire/Gloucestershire Clinical Commissioning Group, Dursley, United Kingdom; ^c^Department of Epidemiology and Public Health, University College London, London, United Kingdom

Dear Editor,

There is increasing interest in referring primary care patients to non-medical sources of support within the community [1]. Commonly referred to as “social prescribing”, this is most often undertaken for mental health, especially for patients with mild-moderate depression for which traditional medical approaches have not been found to be as effective as for more severe depression [2]. One of the popular activities in pilot studies has been encouraging patients to engage in activities that support the uptake of new hobbies, such as groups or societies that involve making music, drawing, handicrafts such as sewing, carpentry, collecting, or model-making. These activities are related to other leisure activities such as volunteering in that they provide distraction, novelty, cognitive stimulation, belongingness as well as enhancing coping skills and agency and (when engaged in as part of a group) provide social support, all of which are positively associated with mental health. But hobbies specifically also provide sensual engagement, self-expression, creativity, and relaxation [3].

In relation to depression, intervention studies have reported benefits from engaging in hobbies such as arts activities [4, 5]. However, they are limited by small sample sizes, short follow-up, and often focus on specific engagement with an initial group involving hobby participation rather than any ongoing home-based hobby engagement that may follow. Larger cross-sectional studies have shown associations between hobbies and lower depressive symptoms [6], while some longitudinal studies have shown associations between specific hobbies and depressive symptoms [7]. However, such studies have treated hobby engagement as static, and it remains unclear whether dynamic changes in hobby engagement are associated with changes in depression over time, and whether such hobbies provide support in the prevention or treatment of depression. Therefore, this study investigated the longitudinal time-varying association between engagement with hobbies and depressive symptoms later in life.

Data came from 8,780 adults aged 50+ from the English Longitudinal Study of Ageing, with biennial measures from 2004/5 to 2016/17 [8]. We used self-reported engagement with hobbies and measured depression using the Centre for Epidemiological Studies Depression scale. We used fixed-effects regression models, which explore within-person variation with individuals serving as their own reference point, compared with themselves over time. So, all time-invariant covariates, such as childhood exposure to hobbies, intelligence, genetics, personality, medical history, and social class, are accounted for, even if unobserved [9]. Further, fixed-effects regression allows us to assess the dynamic relationship between changes in hobbies over time, with changes in depression, whilst controlling for changes in confounding factors (see online suppl. materials for further methods; for all online suppl. material, see www.karger.com/doi/10.1159/000503571).

Of the 8,780 participants, 55.0% were female and the majority (97.7%) were white. Participants had a mean age of 66.9 years (SD 10.1, range 52–99). At baseline, 71.9% reported having a hobby or pastime, and 15.6% were above the threshold for depression using the Centre for Epidemiologic Studies Depression Scale (CES-D). Sample characteristics are provided in online supplementary Table 1.

When controlling for all identified time-varying confounders, taking up a hobby was associated with a decrease in depressive symptoms (coefficient −0.28, 95% CI −0.34 to −0.23) and a 30% lower odds of experiencing depression (OR 0.70, 95% CI 0.64 to 0.76) (Table 1). Whilst around 16% of the association was explained by time-varying demographic and health-related factors, and a further 7% was explained by time-varying reading habits, social engagement, and physical activity, the rest of the association was independent of all of these factors.

Our results were consistently found amongst both men and women, those who were free from depression at baseline, and those who already had depression at baseline. Findings remained robust after sensitivity analyses adjusting for available free time and money, using a broader definition of hobby, using alternative thresholds for depression, including self-reported physician diagnoses of mental illness, and applying time-lagged analyses to explore the direction of association.

We also simulated an intervention whereby participants who did not have a hobby might be referred to take part in a community activity that involved taking up a hobby. First, we restricted the sample to those who were free from depression at baseline and did not have a hobby. For these participants, taking up a hobby was associated with the maintenance of lower levels of depressive symptoms (fully adjusted model coefficient −0.26, 95% CI −0.34 to −0.17) and a 32% lower odds of developing depression (fully adjusted model with the binary outcome coding reversed to show incidence, OR 0.68, 95% CI 0.56–0.83). Second, we restricted the sample to those who had depression at baseline and did not have a hobby. For these participants, taking up a hobby was associated with an improvement in depressive symptoms (fully adjusted model coefficient −0.49, 95% CI −0.69 to −0.28) and a 272% higher odds of recovering from that depression (fully adjusted model with the binary outcome coding reversed to show recovery, OR 2.72, 95% CI 2.09–3.53).

This study has a number of strengths including its large representative sample, repeated longitudinal follow-up, and robust statistical approach. Nevertheless, analyses were limited by the data included in ELSA, so the effects of antidepressant use could not be analysed. However, previous research shows hobby engagement is not altered by taking medication, suggesting that medication would not technically act as a confounder [10]. Further, the CES-D is not a clinical diagnostic tool for depression. However, different thresholds and consideration of self-reported diagnosis did not affect results. Our sample was predominantly white British, so whether results can be generalised to other populations remains to be explored. Finally, it remains unknown whether participants took part in hobbies with others. Analyses showed consistent results when adjusting for level of social interaction, suggesting results were independent of any social engagement that occurred from taking part in hobbies. But whether social hobbies have a stronger association remains to be further explored.

Our findings support the ongoing work of clinicians, psychologists, psychotherapists, and social workers in using social prescribing of hobbies as a supplement to patients' existing care plans (although care should continue to be taken to ensure the suitability for each patient). Further, this study provides support for social prescriber link workers to explore patients' current engagement and willingness to engage in new hobbies as an integral part of a social prescription.

## Statement of Ethics

ELSA receives ethical approval from the National Research Ethics Service, and all participants provided informed consent.

## Disclosure Statement

The authors have no conflicts of interest to declare.

## Funding Sources

The English Longitudinal Study of Ageing was developed by a team of researchers based at the University College London, NatCen Social Research, the Institute for Fiscal Studies, and the University of Manchester. The data were collected by NatCen Social Research. The funding is provided by National Institute of Aging Grant R01AG017644 and a consortium of UK government departments coordinated by the Economic and Social Research Council. D.F. is supported by the Wellcome Trust (205407/Z/16/Z). The funders had no role in the study's design, conduct, or reporting.

## Author Contributions

D.F. and C.O. designed the study. D.F. carried out the analyses and drafted the manuscript. All three authors interpreted the findings, critically appraised the manuscript, and approved it for submission.

## Supplementary Material

Supplementary data

## Figures and Tables

**Table 1:** Longitudinal time-varying associations between having a hobby and depression using fixed effects regression models

	Depressive symptoms (continuous)			Depression (binary)		
	coefficient	95% CI	*P*	OR	95% CI	*P*
*Main analyses*						
Model 1	−0.45	−0.50 to −0.39	<0.001	0.57	0.52 to 0.62	<0.001
Model 2	−0.33	−0.39 to −0.28	<0.001	0.66	0.60 to 0.72	<0.001
Model 3	−0.28	−0.34 to −0.23	<0.001	0.70	0.64 to 0.76	<0.001
Total observations (individuals)	61,460 (8,780)			27,020 (3,860)^a^		

*Subgroup analyses (all using model 3)*						
Men only	−0.29	−0.35 to −0.21	<0.001	0.68	0.59 to 0.79	<0.001
Total observations (individuals)	27,643 (3,949)			10,437 (1,491)^a^		
Women only	−0.28	−0.35 to −0.21	<0.001	0.72	0.64 to 0.81	<0.001
Total observations (individuals)	33,817 (4.831)			16,583 (2,369)^a^		
Free from depression at baseline	−0.24	−0.29 to −0.18	<0.001	0.72	0.63 to 0.82	<0.001
Total observations (individuals)	51,775 (7,401)			17,742 (2,536)^a^		
With depression at baseline	−0.35	−0.49 to −0.22	<0.001	1.38^c^	1.14 to 1.67	0.001
Total observations (individuals)	9,557 (1,379)			9,243 (1,321)^a^		
Free from depression at baseline and no baseline hobbies	−0.26	−0.34 to −0.17	<0.001	0.68	0.56 to 0.83	<0.001
Total observations (individuals)	13,754 (2,008)			5,530 (803)^a^		
With depression at baseline and no baseline hobbies	−0.49	−0.69 to −0.28	<0.001	2.72^c^	2.09 to 3.53	<0.001
Total observations (individuals)	4,154 (613)			4,049 (596)^a^		

*Sensitivity analyses (all using model 3)*						
Additionally adjusting for sufficient time and money	−0.04	−0.05 to −0.03	<0.001	0.71	0.65 to 0.78	<0.001
Total observations (individuals)	61,460 (8,780)			27,020 (3,860)^a^		
Using a broader definition of hobby	−0.04	−0.05 to −0.03	<0.001	0.70	0.63 to 0.77	<0.001
Total observations (individuals)	61,460 (8,780)			27,020 (3,860)^a^		
Using an alternative cut-off CES-D ≥3	−			0.70	0.64 to 0.76	0.001
Total observations (individuals)				38,577 (5,511)^a^		
Additionally including physician diagnoses of depression	_^b^			0.72	0.65 to 0.79	<0.001
Total observations (individuals)				28,091 (4,013)^a^		
Accounting for time-lagged effects using the						
Arellano-Bond estimator	−0.26	−0.34 to −0.19	<0.001	_^d^		
Total observations (individuals)	43,900 (8,780)					

Model 1 automatically accounts for all time-invariant factors even if unobserved (such as sex, ethnicity, educational degree, medical history, prior medication history, genetics, and individual stable risk factors). Model 2 additionally adjusts for time-variant factors that could act as confounders (age, wealth, employment status, eyesight, hearing, chronic pain, frequency of alcohol consumption, whether participants currently smoked, and presence of a chronic physical illness (including cancer, COPD, diabetes, angina, arthritis, or a stroke in the last 2 years). Model 3 additionally adjusts for further time-variant factors that could act as confounders but could potentially lie on the causal pathway (reading a daily newspaper, social engagement, and sedentary behaviours). All results reported show unstandardised fixed effects regression coefficients or odds ratios (OR).

a Lower individual observations as xtlogit drop participants with all positive or all negative outcomes.

b No change to previous analyses as physician diagnosis is separate from depression symptom score.

c Binary outcome reversed to show odds ratio for recovery from depression given depression at baseline.

d Arellano-Bond estimators cannot be computed for binary outcomes.
