# Supplementary material for: Fixed-Effects Analyses of Time-Varying Associations between Hobbies and Depression in a Longitudinal Cohort Study: Support for Social Prescribing?
Source: Psychother Psychosom. 2019 Oct 28;89(2):111–3. doi: 10.1159/000503571 (PMC7158225; doi:10.1159/000503571)
Supplement: Supplementary file 1 — Supplementary data [file pps-0089-0111-s01.docx]

Supplementary Materials

**Participants**

Participants were drawn from the English Longitudinal Study of Ageing (ELSA): a nationally-representative panel study of adults aged 50+ in the UK (1). ELSA was designed as a stratified random sample of private households drawn originally from 1998, 1999 and 2001 Health Survey for England (HSE) (2). The study provides high quality longitudinal data that includes particularly rich data on physical and mental health, social relationships, cognitive function, economic circumstances, and biology. For this study, we used data from wave 2 (2004-2005) as baseline since it included the relevant variables for this analysis. This wave had a response rate of 82% (n=8,780) from core responders, and all were included in these analyses. Participants were then followed up biennially for the following 12 years to wave 8 (2016-2017; a total of 7 waves of data collection), with missing data across waves imputed to preserve the full sample (see ‘statistics’). ELSA receives ethical approval from the National Research Ethics Service and all participants provided informed consent.

**Measures**

Depression was measured using the Centre for Epidemiological Studies Depression (CES-D) scale; a widely used self-report measure of depressive symptoms (3). Analyses specifically involved the 8-item version, which has been found to have comparable psychometric properties to the full 20-item scale (4). In the most recent wave of ELSA, the Cronbach’s alpha for the scale is 0.75. The scale is used to measure general depressive symptoms, and also two specific thresholds have been proposed to denote the presence of depression and validated against standardised psychiatric interviews with older adults: a score of 3 and above (4), and 4 and above (5). For this analysis we showed both the results for depressive symptoms as a continuous outcome and for depression as a binary outcome using the higher cut-off to reduce the risk of false positives. But we conducted sensitivity analyses using the lower cut-off too, given its frequent usage in research.

Participants were asked to self-report through participant questionnaire whether they currently had a hobby or pastime. Hobbies are commonly defined as informal leisure activities that were not done as an individual’s primary occupation, not done for money, and that did not involve substantial levels of physical activity; evaluations have suggested that public perceptions of hobbies match this definition (6,8,9). In the participant interview, self-reports of ‘hobbies’ were distinguished from other leisure activities including cultural engagement, community group membership, physical activity, sports groups and reading newspapers, which were asked about separately alongside the question on hobbies. Specific definitions of hobbies vary, from an exclusive focus on crafts (e.g. playing a musical instrument, drawing, sewing, carpentry and model-making) and collecting (e.g. stamps or postcards) (10), to a broader focus on group activity participation and adult learning (11). As we were unable to ascertain whether participants had included these broader activities when responding to the question on hobbies, in our sensitivity analyses, we expanded our exposure to include self-reported participation in evening or educational classes, arts groups, or music groups measured through other questions in ELSA.

Time-invariant factors (not included in analyses but shown descriptively and considered automatically within unadjusted model 1) included gender (male or female), marital status (married/cohabiting vs single/widowed/divorced), ethnicity (white British vs other), educational qualifications (no educational qualifications; education to age 16/O-levels; education to age 18/A-levels; degree/higher qualification). Time-variant factors that could act as confounders (included in analyses in model 2) included age (in categories 50-55, 56-60, 61-65, 66-70, 71-75, 76+), total non-pension wealth (quintiles) (12), employment status (working full time vs working part time vs not working), eyesight (excellent/very good/good vs fair/poor), hearing (excellent/very good/good vs fair/poor), chronic pain (none/mild vs moderate/severe), frequency of alcohol consumption (1-2 days a week, 3-4 days a week, 5-6 days a week or daily), whether participants currently smoked, and presence of a chronic physical illness (including cancer, COPD, diabetes, angina, arthritis or a stroke in the last 2 years). In order to test whether having a hobby was a proxy for broader cognitive stimulation, social interaction or helping to keep people more active, additional analyses considered whether participants read a newspaper, how frequently they had social contact with friends, family or wider relatives (either face-to-face, via telephone or via writing/emailing), and whether they led a sedentary lifestyle (mild, moderate or physical activity less than once a week) as time-varying covariates.

**Statistics**

Analyses were carried out using Stata 14 (StataCorp, College Station, TX). A total of 27.6% of data were missing at random across the 7 waves so we used multiple imputation by chained equations to generate 50 imputed datasets, providing a full sample size of 8,780 across all waves. Results using imputed data were materially no different from results using raw data, so we present results from imputed data for greater statistical power.

We used fixed-effects regression, which differs from other regression techniques as it explores within-person variation with individuals serving as their own reference point, compared with themselves over time. So all time-invariant (stable) covariates, are accounted for, even if unobserved (13). This approach is advantageous as individual stable characteristics such as childhood exposure to hobbies, intelligence, genetics, personality, medical history and social class could confound associations with depression. But as individuals are compared with themselves, such bias cannot affect results. Additionally, having a hobby can vary over time, as can depression, and both can be affected by time-varying confounders. Fixed-effects regression allows us to analyse these time-varying associations. So in this analysis we were able to assess the relationship between changes in hobbies across each of the seven waves, with changes in depression across each of the seven waves, whilst controlling for changes in confounders across each of the seven waves.

The basic model for depression (CES-D) can be expressed as follows:

$${CESD}_{it}={\beta_{0t}+ \beta_{1}H}_{it}+ \beta_{2}T_{it}+\alpha_{i}+ \varepsilon_{it}$$

where CESD_it_ is a measure of individual $i$'s depression score at time $t$, $\alpha_{i}$ is unobserved time invariant confounding factors, H is whether an individual $i$had a hobby at time $t$, and T is measured time-varying confounding factors. Time-varying confounders were identified by using directed acyclic graphics (DAGs) (14). Data were strongly balanced. A Hausman test was used to confirm the selection of a fixed effects over a random effects model. The modified Wald test for group-wise heteroscedasticity was significant (indicating the presence of heteroscedasticity) so sandwich estimators were applied. Coefficients for all years were not jointly equal to zero, so time-fixed effects were included in the model. We also used linear dynamic panel-data estimators (Arellano-Bond) which uses feedback from past outcomes to predict current exposure and outcomes, helping to identify the direction of association to reduce the risk of reverse causality, thereby helping us to make inferences on causality from these findings. When running the Arellano-Bond estimator, tests for zero autocorrelation and over-identifying restrictions both showed that the model assumptions were satisfied.

In subgroup analyses, we stratified our analyses by sex and baseline depression. We also simulated an intervention. We restricted the sample to participants who did not have a hobby at baseline and either (i) did not have depression at baseline, or (ii) had depression at their baseline and explored whether taking up a hobby was associated either with (i) a lower chance of developing depression, or (ii) a higher chance of recovery from depression.

Further sensitivity analyses tested assumptions. We considered that individuals who did not engage in hobbies might not have the time or financial resources to do so, so we re-ran analyses including questions on whether participants felt they had enough time to do everything they wanted in their lives or too little money to treat themselves from time to time. As some participants may not have considered certain activities as hobbies even though they fell under our definition, we expanded our exposure to include self-reported participation in evening or educational classes, arts groups, or music groups, which can all provide participants with the opportunity in which to undertake hobbies. To test the assumption of a threshold of 4+ on the CES-D scale, the alternative cut-off of 3+ used for identifying broader cases of depression was applied (20). As data were only gathered every 2 years, it is possible that some participants experienced a depressive episode in between data collection but recovered prior to the next data collection wave, so analyses may underestimate the incidence of cases over the follow-up period. So analyses were re-run using a combined index of (i) whether participants were above the threshold for depression on the CES-D scale, or (ii) whether they self-reported a physician diagnosis of depression since the last data collection wave. This produced a further 76 cases (equivalent to an additional 0.9% of participants experiencing depression). Finally, as fixed-effects regression models are static and have a strict exogeneity assumption, they do not make use of observable information from previous time periods. So linear dynamic panel-data models using the Arellano-Bond estimator were applied, which relax the exogeneity assumption and allow the inclusion of a time-lagged outcome, using the value of the dependent variable in the previous period as a predictor for the current value of the dependent variable (15). This can help to confirm the direction of association. Sensitivity analyses were also run on all sub-group analyses and did not materially affect results so results from the imputed data set are presented here.

**Results**

Participants who had a hobby at baseline were more likely to be male, have higher educational qualifications, be younger in age, married, of higher wealth, still working part- or full-time, be more socially engaged and lead healthier lifestyles. Pearson’s correlations showed an association between engaging in hobbies and level of depressive symptoms -0.17 (p<.001), suggesting 3% of the variation in depressive symptoms was explained by engagement in hobbies.

**Supplementary Table 1: Sample characteristics and chi-square test comparing the characteristics of individuals with and without a hobby**

| Characteristic | N (%) | Has a hobby (71.9%) | Does not have a hobby (28.1%) | p |
| --- | --- | --- | --- | --- |
| Time-invariant characteristics ^a^ |  |  |  |  |
| Sex, % female | 4,831 (55.0%) | 53.7% | 58.1% | <.001 |
| White, % | 8,574 (97.7%) | 98.8% | 97.4 | <.001 |
| Educational attainment, % |  |  |  | <.001 |
| No qualifications / basic qualifications | 3,878 (44.2%) | 34.6% | 60.8% |  |
| GCSE / O-level / qualification at age 16 | 1,460 (16.7%) | 19.1% | 13.1% |  |
| A-levels / higher education / qualification at age 18 | 2,375 (27.1%) | 30.9% | 20.5% |  |
| Degree / further higher qualification | 1,057 (12.1%) | 15.4% | 5.6% |  |
| Time-varying characteristics (stated at baseline) |  |  |  |  |
| Age, % |  |  |  | <.001 |
| 50-55 | 1,083 (12.3%) | 12.9% | 12.1% |  |
| 56-60 | 1,859 (21.2%) | 23.4% | 18.6% |  |
| 61-65 | 1,399 (15.9%) | 17.5% | 14.1% |  |
| 66-70 | 1,386 (15.8%) | 17.0% | 15.2% |  |
| 71-75 | 1,203 (13.7%) | 13.3% | 14.5% |  |
| 76+ | 1,850 (21.1) | 15.9% | 25.5% |  |
| Married/cohabiting, % | 5,955 (67.8%) | 73.2% | 64.1% | <.001 |
| Lowest wealth quintile, % | 1,733 (20.0%) | 13.9% | 27.9% | <.001 |
| Working full- or part-time, % | 2,764 (31.7%) | 34.9% | 28.3% | <.001 |
| Fair/poor eyesight, % | 1,285 (14.6%) | 9.9% | 19.6% | <.001 |
| Fair/poor hearing, % | 1,933 (22.0%) | 19.3% | 26.3% | <.001 |
| Frequency of drinking alcohol, % |  |  |  | <.001 |
| Less than once a week | 4,143 (47.2%) | 36.7% | 50.1% |  |
| Once or twice a week | 1,941 (22.1%) | 25.7% | 22.9% |  |
| 3 or 4 times a week | 895 (10.2%) | 12.6% | 8.6% |  |
| 5 or more times a week | 1,801 (20.5%) | 25.1% | 18.4% |  |
| Smoker, % | 1,329 (15.2%) | 12.5% | 19.6% | <.001 |
| Has a chronic health condition, % | 3,220 (36.7%) | 33.8% | 41.0% | <.001 |
| Has chronic pain, % | 2,418 (27.5%) | 23.7% | 34.9% | <.001 |
| Reads a daily newspaper, % | 5,181 (59.0%) | 68.3% | 66.3% | .082 |
| Lowest quartile for social interaction, % | 2,111 (24.0%) | 12.0% | 20.8% | <.001 |
| Leads a sedentary lifestyle, % | 749 (8.6%) | 5.1% | 12.6% | <.001 |

***^a^*** *Shown here for descriptive purposes but not included in analysis*

*^b^ Wealth divided into quintiles so not reported here*

**References**

1. Steptoe A, Breeze E, Banks J, Nazroo J. Cohort Profile: The English Longitudinal Study of Ageing. Int J Epidemiol. 2013 Dec;42(6):1640–8.

2. Steptoe A, Breeze E, Banks J, Nazroo J. Cohort Profile: The English Longitudinal Study of Ageing. Int J Epidemiol. 2013 Dec 1;42(6):1640–8.

3. Radloff LS. The CES-D Scale: A Self-Report Depression Scale for Research in the General Population. Appl Psychol Meas. 1977 Jun 1;1(3):385–401.

4. Turvey CL, Wallace RB, Herzog R. A revised CES-D measure of depressive symptoms and a DSM-based measure of major depressive episodes in the elderly. Int Psychogeriatr. 1999 Jun;11(2):139–48.

5. Steffick DE. Documentation of affective functioning measures in the Health and Retirement Study. Ann Arbor MI HRS Health Work Group. 2000;

6. Neulinger J. The psychology of leisure: Research approaches to the study of leisure. Springf I. 1974;11:295–306.

7. Adams KB, Leibbrandt S, Moon H. A critical review of the literature on social and leisure activity and wellbeing in later life. Ageing Soc. 2011 May;31(4):683–712.

8. Stebbins RA. Serious Leisure: A Conceptual Statement. Pac Sociol Rev. 1982 Apr;25(2):251–72.

9. Gelber SM. Hobbies: Leisure and the culture of work in America. Columbia University Press; 1999.

10. Daily LZ. Towards a definition of “hobby”: An empirical test of a proposed operational definition of the word hobby. J Occup Sci. 2018 Jul 3;25(3):368–82.

11. Stebbins RA. The liberal arts hobbies: A neglected subtype of serious leisure. Loisir SociétéSociety Leis. 1994;17(1):173–186.

12. Banks J, Karlsen S, Oldfield Z. Socio-economic position. In: Marmot M, Banks J, Blundell R, Lessof C, Nazroo J, editors. Health, Wealth and Lifestyles of the Older Population in England. Institute for Fiscal Studies, London; 2003. p. 71–125.

13. Allison PD. Fixed effects regression models. Vol. 160. SAGE publications; 2009.

14. Shrier I, Platt RW. Reducing bias through directed acyclic graphs. BMC Med Res Methodol. 2008 Oct 30;8:70.

15. Gunasekara FI, Richardson K, Carter K, Blakely T. Fixed effects analysis of repeated measures data. Int J Epidemiol. 2014 Feb 1;43(1):264–9.
